# Porcine Gammaherpesviruses in Italian Commercial Swine Population: Frequent but Harmless

**DOI:** 10.3390/pathogens10010047

**Published:** 2021-01-07

**Authors:** Giovanni Franzo, Michele Drigo, Matteo Legnardi, Laura Grassi, Maria Luisa Menandro, Daniela Pasotto, Mattia Cecchinato, Claudia Maria Tucciarone

**Affiliations:** Department of Animal Medicine, Production and Health (MAPS), University of Padua, 35020 Legnaro (PD), Italy; michele.drigo@unipd.it (M.D.); matteo.legnardi@phd.unipd.it (M.L.); laura.grassi.2@phd.unipd.it (L.G.); marialuisa.menandro@unipd.it (M.L.M.); daniela.pasotto@unipd.it (D.P.); mattia.cecchinato@unipd.it (M.C.); claudiamaria.tucciarone@unipd.it (C.M.T.)

**Keywords:** *Suid gammaherpesvirus*, PLHV, molecular epidemiology, Italy, co-infection, disease

## Abstract

Differently from alpha- and betaherpesviruses affecting swine, interest in the recently discovered *Suid gammaherpesvirus 3*, *Suid gammaherpesvirus 4*, and *Suid gammaherpesvirus 5*, also known as porcine lymphotropic herpesviruses (PLHV-1, PLHV-2, and PLHV-3), has largely focused on their role as potential zoonotic agents in cases of xenotransplantation. However, their role as primary pathogens of swine or as co-factors for other lymphotropic infections has essentially been neglected. The present study aims at filling this gap, evaluating the association between PLHVs infection and different clinical conditions and/or porcine circovirus (PCV) co-infection. One hundred seventy-six samples were obtained from different animals located in a high-density pig area of Northern Italy in the period 2017–2020. The presence of PLHVs and PCVs was tested and quantified by specific real-time PCR: PLHVs were widespread among pigs (PLHV-1, PLHV-2, and PLHV-3 prevalence was 28.97%, 10.79%, and 4.54%, respectively) and detected in all considered tissues and clinical conditions. Frequent co-infections were also observed among PLHVs and with PCVs, although a significant association was not detected with the exception of a positive interaction between PLHV-1 and PLHV-3, and a negative one between PLHV-2 and PCV-2. Significantly, no association between PLHVs, alone or in co-infection, emerged with any of the considered clinical signs, their frequency being comparable between healthy and diseased animals. Based on these pieces of evidence and despite their high prevalence, PLHVs’ relevance for the swine industry appears negligible, either as primary pathogens or as predisposing factors for circovirus-induced diseases.

## 1. Introduction

Domestic pigs are affected by two main herpesviruses—pseudorabies virus (PRV) (species *Suid alphaherpesvirus 1*), a member of the *Varicellovirus* genus, and porcine cytomegalovirus (PCMV) ((species *Suid betaherpesvirus 2*), a member of the *Roseolovirus* genus—which cause overt clinical signs and associated economic losses [1,2].

More recently, three new porcine lymphotropic herpesviruses (PLHV-1, PLHV-2, and PLHV-3) [3,4], formally classified into *Suid gammaherpesvirus 3, Suid gammaherpesvirus 4*, and *Suid gammaherpesvirus 5*, have been identified and assigned to the genus *Macavirus* (https://talk.ictvonline.org/taxonomy/) because of their phylogenetic clustering with other members of this group. Since their discovery, knowledge on their epidemiology has remained extremely limited. Transmission is thought to occur mainly horizontally [5], even if the detection of PLHVs in caesarean-derived piglets suggests that vertical transmission, although rare, is possible [6]. Despite the limited number of studies performed on these viruses in recent decades, they seem widespread in domestic and wild suid populations as their circulation has been reported in different countries with variable prevalence [5]. In Germany, PLHV-1, PLHV-2, and PLHV-3 were, respectively, found in 78%, 41%, and 59% of the lung and 59%, 26%, and 62% of the investigated spleen samples [4]. Similarly, a high detection frequency (i.e., 80%, 20%, and 65% for PLHV-1, -2, and -3, respectively) was identified in a small set of 20 Italian samples [4]. Comparable pieces of evidence were reported from Spain, France, USA, and Ireland [4,5,7]. 

Notwithstanding the wide circulation in the swine population, the clinical implications seem marginal and have never been thoroughly investigated. Interest has been monopolized by the risk connected to xenotransplantation. PLHV-1 was associated with posttransplant lymphoproliferative disease in the miniature pig experimental model following allogenic hematopoietic stem cell transplantation [8,9]. However, the clinical significance of PLHVs infection in traditional intensive pig farms has been substantially neglected. 

The evidence on the biological cycle of PLHVs is extremely limited. However, lymphoid tissues and cells appear the main target of viral replication.

Viral genome presence was detected in several cells and tissues including peripheral blood mononuclear cells (PBMCs), tonsils, spleen, liver, kidney, aorta, salivary gland, and lung [4,6,7,10]. Currently, PLHVs have not been successfully propagated in cell cultures, although PLHV-3 has been detected in a persistently infected porcine B cell line L23 [4]. 

Because of their tropism, an effect on the immune system was speculated and the interaction with porcine circovirus 2 (PCV-2) in the pathogenesis of postweaning multisystemic wasting syndrome (PMWS) was evaluated by McMahon et al. (2006) [7]. While no association between PMWS and PLHVs was established, the limited number of affected animals, combined with the multifactorial nature of the disease, could have decreased the power of the study. Moreover, the lack of information on PCV-2 and PLHVs within-host viral titers could hinder more subtle associations among these viruses. More generally, the frequency and magnitude of PLHVs infection have never been investigated in intensively raised commercial pigs suffering from different clinical conditions. 

Another porcine circovirus, porcine circovirus 3 (PCV-3), sharing several biological features with PCV-2, has been recently identified and has become the focus of intense research activity [11,12]. Nevertheless, its actual clinical relevance and pathogenesis, including interaction with other co-infections, is largely unknown and debated [11,13]. Whether lymphotropic agents like PLHVs can actually modulate PCV-3 virulence has never been investigated.

The present work aims at filling this gap by testing samples obtained from a high-density pig area of Northern Italy for both routine monitoring and diagnostic purposes and evaluating the association with clinical diseases and circoviruses co-infection occurrence.

## 2. Results

### 2.1. Infection Frequency and Viral Genome Quantification

Fifty-one (28.97%), 19 (10.79%), and 8 (4.54%) samples tested PLHV-1-, PLHV-2-, and PLHV-3-positive, while PCV-2 and PCV-3 were detected in 72 (40.90%) and 14 (7.95%) samples, respectively. Co-infections were identified among PLHVs and circoviruses (Table 1).

However, no statistically significant association between infections was found, with the only exception being PLHV-1/PLHV-3 and PLHV-2/PCV-2, featured by an excess and dearth of co-infections compared to what was expected by chance, respectively. Accordingly, a significant positive correlation (Rho = 0.27; *p* < 0.001) between PLHV-1 and PLHV-3 genome copy number/DNA µL was observed, while a negative one (Rho = −0.18; *p* = 0.017) was observed between PLHV-2 and PCV-2. 

### 2.2. Association with Different Clinical Conditions and Sample Types

No association was detected between PLHVs infection and clinical signs, since PLHVs were detected at a comparable frequency and genome copy number/DNA µL both in healthy subjects and in subjects with different clinical conditions (Table 2).

**Figure 1:**
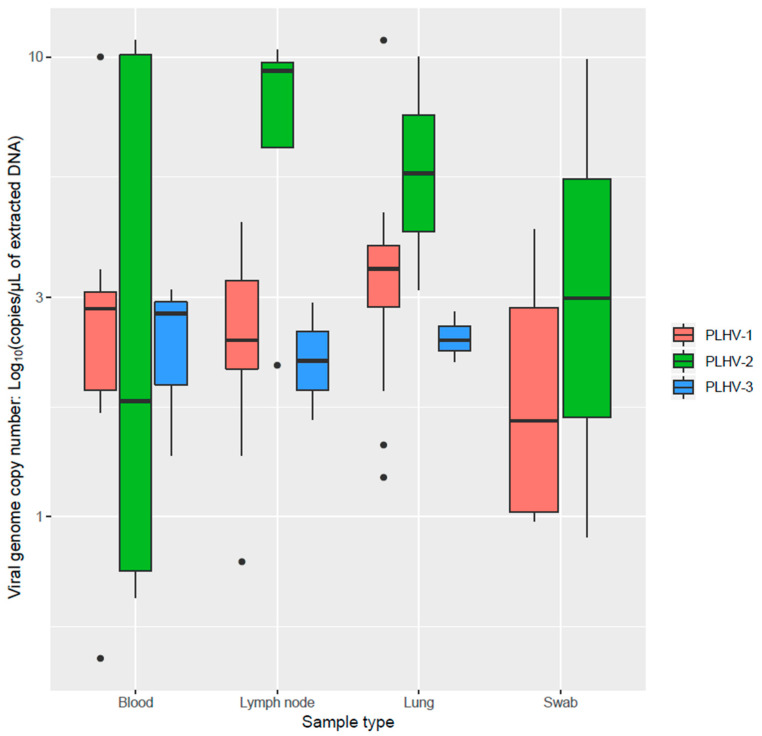
Boxplot reporting the viral genome copy number/DNA µL of different PLHVs (color-coded) in the considered matrices.

PLHVs were detected in all considered sample types except for PLHV-3, which was not detected in nasal swabs. PLHV-1 was found at a significantly higher frequency in lymph nodes and at a lower one in blood samples (*p* < 0.001). However, a higher genome copy number/DNA µL was also identified in blood and lungs compared to lymph nodes (Figure 1). A deviation from what expected by chance alone was observed for PLHV-2 also (*p* = 0.047), for which a trend toward a higher frequency in lymph nodes and blood was present.

## 3. Discussion

PLHVs were identified more than 20 years ago and have attracted certain interest as a potential zoonotic agent in cases of xenotransplantation. On the other hand, the epidemiological and clinical features of these viruses, both alone and in co-infection, have been only marginally investigated. 

The present study confirms the high frequency of PLHVs infection in domestic swine populations. Comparable to the few available studies, PLHV-1 was the most prevalent. However, differently from Chmielewicz et al. (2003) [4], PLHV-2 was detected at a higher frequency than PLHV-3. Additionally, an overall lower prevalence was herein demonstrated. Therefore, local variations in PLHVs epidemiology are likely, even though the underlying causes are difficult to establish. Italian swine farming has some peculiarities compared to other countries since pigs are raised up to 160 kg for the production of cured ham. The slower population turnover, higher average age, and unavoidable management complications (e.g., difficulties in all-in all-out, flow management, etc.) could affect PLHVs epidemiology [14]. However, dedicated comparative studies should be performed to confirm this hypothesis. Alternatively, most of the previous studies were based on selected matrices, particularly spleen and peripheral blood mononuclear cells (PBMCs), which could represent more suited target tissues. Accordingly, when PLHVs’ detection frequency was evaluated in different matrices, PLHV-1 frequency in blood was significantly lower, further confirming the primary lymphoid tropism of at least some of the considered viruses. However, a higher genome copy number was detected in blood samples. Although surprising, a relationship with different infectious phases could be speculated, with a higher viremia peak followed by persistence in lymphoid tissues. Unfortunately, only one tissue was available for each subject. Therefore, comparison of the viral load distribution within-animal was not possible. Nevertheless, all tissues tested positive, including lungs and respiratory swabs, demonstrating a broad viral tropism and strengthening horizontal transmission as the most likely infection route [5], potentially through salivary or respiratory secretions. These results are in agreement with the experimental findings of Tucker et al. (2003), which demonstrated PLHV presence in tonsils [9].

Frequent co-infections were identified. Interestingly, a higher than expected co-infection occurrence was proven between PLHV-1 and PLHV-3, which might suggest an interaction between these two PLHVs, as confirmed by the positive correlation between their viral amount. 

A relationship with PCV-2, whose biological cycle and pathogenesis are deeply linked to the immune system [15,16], has previously been investigated and no contribution of PLHVs to PMWS development was identified [7]. However, the limited number of involved subjects, especially with clinical signs, limited the confidence of such results. The present study confirms the absence of positive associations between PLHVs and PCV-2. Similarly, no statistically significant association was identified with PCV-3 infection. A negative correlation between PCV-2 and PLHV-2 was proved instead. Interestingly, PLHV-2 was also the only undetected PLHV in co-infection with PCV-2 in McMahon et al. (2007) [7]. A potential competition for the cell pool could thus be hypothesized, although the actual mechanism is currently unknown. 

Overall, no association between PLHVs, alone or in co-infection, emerged with any of the considered clinical signs, their frequency being comparable between healthy and diseased animals. Based on these pieces of evidence and despite their high prevalence, the relevance of PLHVs for the swine industry appears negligible, either as primary pathogens or predisposing factors for circovirus-induced diseases.

Further investigations will be essential to establish whether porcine macaviruses could favor other pathogens or if other co-factors are required for their pathogenetic expression.

## 4. Materials and Methods 

### 4.1. Samples

A total of 176 samples were collected from 139 farms located in a high-density pig area of Northern Italy in the period 2017–2020. Samples were delivered to the Laboratory of Infectious Diseases of the Dept. of Animal Medicine, Production and Health (MAPS) of the University of Padua for routine monitoring activity or diagnostic purposes in the presence of clinical signs. Therefore, no ethical approval was required according to national legislation. More in detail, 101 samples were delivered in the context of monitoring activities while the remaining were obtained from 43 subjects with systemic diseases, 24 with respiratory signs, and 8 with reproductive disorders. All samples were assigned to one of these categories based on a combination of clinical evaluation and/or necropsy results (when samples originated from dead subjects) performed by the field veterinarians or by reference laboratories. When available, reports of histopathology and collateral tests were also accounted for in the final classification. 

Sixty-eight blood samples, 68 lungs, 26 lymph nodes, and 18 nasal swabs were included in the study. Two-hundred microliters of serum were directly extracted while tissues were preliminary mechanically homogenized in PBS (ratio tissue/PBS = 1 g/10 mL) and centrifuged for 5 min at 2000× *g*. Swabs were resuspended in 1 mL of PBS and thoroughly vortexed. Two-hundred microliters of tissue or swab supernatant were used for DNA extraction.

DNA was extracted using the DNeasy Blood & Tissue kits (Qiagen) according to the manufacturer’s instructions. Before extraction, the samples were spiked with the exogenous internal control (IC) provided in the QuantiNova Pathogen + IC kit (Qiagen).

### 4.2. Real-Time PCR Assays

PLHVs diagnosis and titration were performed using primers and probes described by McMahon et al. (2006) [7].

A multiplex reaction was validated for the detection of PLHV-1, PLHV-2, and PLHV-3, plus the internal control, using the QuantiNova Pathogen + IC kit (Qiagen). 

A plasmid including the regions amplified by selected primers was synthesized at GenScript and used as the positive control. The plasmid was resuspended in biology grade water and the plasmid copy number/µL was calculated based on the length using the DNA Copy Number and Dilution Calculator webtool (Thermo Fisher). A ten-fold dilution ranging between 1 × 10^8^ and 1 × 10^0^ copies/µL was used for assay optimization and performance evaluation. For this purpose, different reagent concentrations and thermal protocols were evaluated. The final protocol, allowing the detection of up to 10 copies/µL for each virus, was the following:

1X QuantiNova Pathogen Master Mix, 0.8 μM of forward and reverse primers, and 0.25 μM of each probe (except for PLHV-2, for which a 0.4 μM probe concentration was used). 1X QuantiNova IC Probe Assay was also included in each reaction. Biology grade water was added up to a final value of 10 µl. The following thermal protocol was used: 2 min at 95 °C followed by 45 cycles at 95 °C for 5 s and 55 °C for 30 s. Fluorescence was acquired at the end of the extension phase. A plasmid-based standard curve, created with the optimized protocol, was used for viral genome copy number quantification of the analyzed samples, expressed as viral genome copy number for µL of extracted DNA (viral genome copy number/DNA µL).

PCV-2 and PCV-3 diagnosis was performed as described by Franzo et al. (2020) [14], and Franzo et al. (2018) [17], respectively.

### 4.3. Statistical Analysis

The association between viral infections was evaluated through Fisher’s exact test while the difference in viral genome copy number/DNA µL was assessed using the Kruskal–Wallis test followed by the Mann–Whitney test, with Bonferroni correction for multiple comparisons. The Spearman’s rank correlation coefficient between viral genome copy number/DNA µL pairs was also evaluated. The statistical significance level was set at α = 0.05. All analyses were performed in R.

## Figures and Tables

**Table 1:** Number (upper matrix triangle) and frequency (lower matrix triangle) of co-infections between virus pairs. The number and percentage of co-infections for each virus are reported in the last column and row, respectively.

	PLHV-1	PLHV-2	PLHV-3	PCV-2	PCV3	TOTAL
PLHV-1		5	7	25	5	42
PLHV-2	2.84%		0	3	2	5
PLHV-3	3.97%	0%		4	1	5
PCV-2	14.20%	1.70%	2.27%		5	5
PCV-3	2.84%	1.13%	0.56%	2.83%		12
TOTAL	23.85%	2.83%	2.83%	2.83%	6.82%	69/176 (39.2%)

**Table 2:** Ratio and percentage (between brackets) of viral detections in pigs with different clinical status.

	Healthy	Systemic	Respiratory	Reproductive
PLHV-1	25/101 (24.75%)	17/43 (39.53%)	8/24 (33.33%)	1/8 (12.5%)
PLHV-2	8/101 (7.92%)	8/43 (18.6%)	3/24 (12.5%)	0/8 (0%)
PLHV-3	4/101 (3.96%)	2/43 (4.64%)	1/24 (4.16%)	1/8 (12.5%)
PCV-2	42/101 (41.58%)	21/43 (48.83%)	7/24 (29.16%)	2/8 (25%)
PCV-3	7/101 (6.93%)	5/43 (11.62%)	1/24 (4.16%)	1/8 (12. 5%)

## Data Availability

The data presented in this study are available in the manuscript text.
